# Phenotyping date palm varieties via leaflet cross-sectional imaging and artificial neural network application

**DOI:** 10.1186/1471-2105-15-55

**Published:** 2014-02-24

**Authors:** Vladimir Arinkin, Ilya Digel, Dariusz Porst, Aysegül Temiz Artmann, Gerhard M Artmann

**Affiliations:** 1 Institute for Bioengineering (IFB), Aachen University of Applied Sciences, Heinrich-Mussmann-Str. 1, 52428 Juelich, Germany

**Keywords:** Artificial neural network, Backpropagation algorithm, Fluorescence microscopy, Cultivars, Date palm leaf, Vascular bundles, Phenotyping

## Abstract

**Background:**

True date palms (*Phoenix dactylifera* L.) are impressive trees and have served as an indispensable source of food for mankind in tropical and subtropical countries for centuries. The aim of this study is to differentiate date palm tree varieties by analysing leaflet cross sections with technical/optical methods and artificial neural networks (ANN).

**Results:**

Fluorescence microscopy images of leaflet cross sections have been taken from a set of five date palm tree cultivars (*Hewlat al Jouf, Khlas, Nabot Soltan, Shishi, Um Raheem*). After features extraction from images, the obtained data have been fed in a multilayer perceptron ANN with backpropagation learning algorithm.

**Conclusions:**

Overall, an accurate result in prediction and differentiation of date palm tree cultivars was achieved with average prediction in tenfold cross-validation is 89.1% and reached 100% in one of the best ANN.

## Background

We may ask ourselves why care about date palms (*Phoenix dactylifera*)? The simple answer is: This tree and its fruits were and are important nutrition for humans living in tropical and subtropical countries [1]. The total number of date palm trees in 2001 was about 100 million, distributed over 30 countries producing between 2.5 and 5 million tonnes of fruit per year [2]; the FAO [3] estimated the fruit production to be 7.5 million tonnes for 2010. Interest in the differentiation of date palm cultivars is very great, since high fruit quality and quantity are desired and offshoot leaves of different cultivars look alike to a great extent. Early recognition of cultivar and gender is particular important, due to huge expenses for the growth of at least 8–10 years old trees before they start to bear fruit and their cultivar can be confirmed [4]. As for culturing dates in modern times, offshoots are cut off from mother plants, put in pure sand and watered every day. After 12–15 years, female trees produce fruits differing a lot in quality and quantity. Nowadays tissue culture methods could be used to clone date palms, but there are relatively high chances for spontaneous mutations leading to genotype (and phenotype) changes [5].

The general problem of phenotype description has begun from Wilhelm Johannsen in 1911 [6] by defining the phenotype term, and currently experience a huge agricultural interest in a machine learning based and automate acquisition of phenotypic traits [7-9]. In the date palm agriculture there is a need for early confirmation of a cultivar due to high genetic diversity [10], where machine vision characterisation of a plant’s cultivars can be used to support subjective human observations. To achieve statistically reliable data with the help of modern technology while performing a realistic amount of measurements, the methods used need to be robust and effective. Many phenotype-oriented techniques for date palm cultivars differentiation such as analysis of extracts of fruits and leaves with SDS-PoroPAGE [11], RP-HPLC [12], as well as description of vegetative and reproductive traits [13-15], growth, flowering and yield characters [16] have been reported to be successful. Additionally for other plant cultivars, RP-HPLC/Mass spectrometry [17] and capillary zone electrophoresis [18] techniques have been used. Unfortunately predictive models which would open up easy possibilities for practical applications have not been used in the above-mentioned works. A good example of such application would be work of Wu et al. [19].

Along with phenotype analyses, genotyping-oriented techniques such as genetic fingerprinting by using random amplified polymorphic DNA (RAPD) markers and inter simple sequence repeat (ISSR) markers [20,21] or analysis of leaflet isozymes expression as a genetic marker [22-24] have been used to study the genetic diversity of date palm cultivars. Although the results achieved with these techniques are very good, our intent has been to test the feasibility of approach with focus on phenotypic features and a future possible field application.

**Figure 1:**
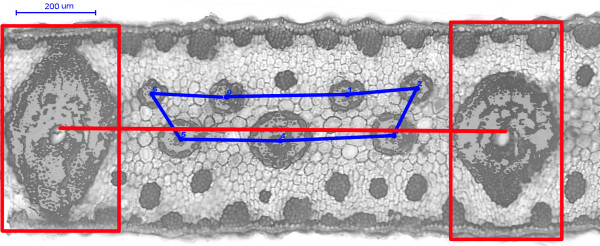
The DAPI-BP fluorescence image of the date palm leaflet cross section, where red line in the middle – baseline, blue line connecting centres of MnVBs – "the shortest pathway" and two red rectangles are fitting MjVBs.

Being vascular plants, date palm trees have a vascular system for transport of water and nutrients as well as for drawing back of waste and produced substances. This vascular system is represented by vascular bundles, which are present in two sizes in date palm leaves: minor vascular bundles (MnVB) and major vascular bundles (MjVB) (see Figure 1). Variability in the distribution patterns of MnVB and shape alteration of MjVB have been observed among cultivars (see Additional files 1, 2, 3, 4). For this reason fluorescence images of leaflet cross sections have been obtained and then processed for classification with an artificial neural network. It is the aim of this study to phenotype date palm varieties via leaflet cross-sectional imaging and artificial neural network application.

## Materials and methods

### Date palm leaves

Samples have been collected from trees of the National Date Palm Research Centre, Saudi Arabia. Leaflets have been collected from the middle part of a pinnae area (an upper part) of the date palm leaf blade. The trees have been growing in similar conditions in the same area.

Leaflets of date palm leaves have been carefully washed with regular warm (35-40°C) water to remove dirt, then washed with room-temperature (25°C) deionized water and wiped with soft cellulose tissues. Leaflets are stored further under nitrogen gas atmosphere (Quality 5.0, ≥ 99.999% pure) to protect them from degradation by aerobic microorganisms and oxidation.

### Fluorescence microscopy

In order to obtain a cross section of a date palm leaflet, it was first precooled (4°C) and fixed with paraffin wax (Roti®-Plast (melting point 56-58°C) from Carl-Roth GmbH + Co. KG, Germany) in a histological sample holder. A 40 μm thick cross section was produced using a microtome (R Jung AG Heidelberg, Germany) and then placed with isotonic 0.9% NaCl (from Carl-Roth GmbH + Co. KG, Germany) water solution on a microscope slide and then covered with a cover glass.

For the acquisition of fluorescence images, a Keyence BZ-8100E fluorescence microscope (Keyence Corp., Osaka, Japan) equipped with a true colours CCD sensor (2/3”, 1.5 megapixels) was used. The following three filters sets (excitation, absorption) were used: DAPI-BP (320-400 nm, 410–510 nm), GFP-BP (430–510 nm, 485–585 nm), Texas-Red (520–600 nm, 570–690 nm) together with a zoom objective CFI Plan Apo VC 20X (Nikon Corp., Tokyo, Japan).

### Image pre-processing

**Figure 2:**
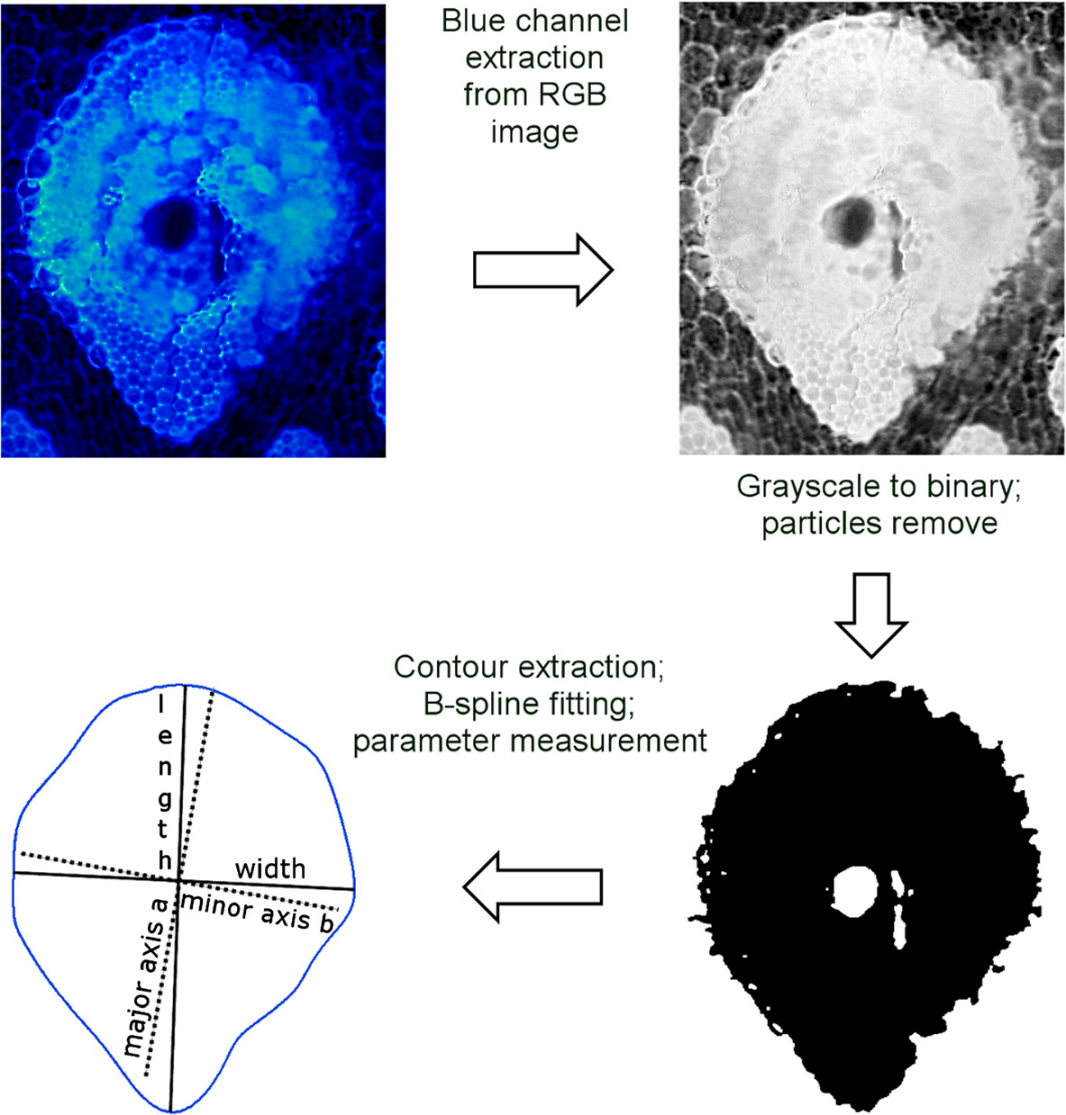
Outline of image processing for the MjVB shape measurement.

Only the images obtained with the DAPI-BP filter have been used for analysis due to their high contrast for vascular bundles. For image pre-processing and feature extraction, two custom-made software based on LabVIEW development environment (National Instruments Corp., Austin, USA) were used, first one for measuring a MnVB distribution and second one for defining a MjVB shape. For the MnVB distribution measurement the blue channel from a DAPI-BP fluorescence RGB image was extracted for simpler handling, see Figure 2. For the definition of a MjVB shape the extracted blue channel was further processed with a brightness and contrast adjustment completed with a threshold conversion to a binary image (pixel values 0/1). After application of “IMAQ particle remove filter 3” filter from LabVIEW development environment to remove particles, the contour of the object was extracted. In order to minimize the influence of hair-like structures on the contour it has been fitted with a set of B-spline curves (15 to 20 curves) and used further on throughout the whole measurement.

### Feature extraction

The LabVIEW-based program for the feature extraction from fluorescence cross-section images works in a semi-automatic mode.

1) For characterisation of a MnVB distribution the following parameters have been introduced:

• *Number of MnVBs between two MjVBs*

•

$$\mathrm{Ratio}=\frac{\sum \left(\mathrm{distances}\;\mathrm{between}\;\mathrm{baseline}\;\mathrm{and}\;\mathrm{MnVB}\;\mathrm{centres}\right)}{\mathrm{length}\;\mathrm{of}\;\mathrm{baseline}}$$

•

$$\mathrm{Ratio}2=\frac{\sum \left(\mathrm{distances}\;\mathrm{between}\;\mathrm{centre}\;\mathrm{of}\;\mathrm{baseline}\;\mathrm{and}\;\mathrm{MnVB}\;\mathrm{centres}\right)}{\mathrm{length}\;\mathrm{of}\;\mathrm{baseline}}$$

•

$$\mathrm{Salesman}\;\mathrm{Ratio}\;=\;\frac{\mathrm{the}\;\mathrm{shortest}\;\mathrm{pathway}\;\mathrm{to}\;\mathrm{visit}\;\mathrm{all}\;\mathrm{MnVBs}}{\mathrm{length}\;\mathrm{of}\;\mathrm{baseline}}$$

The baseline is defined as a line between the centres of two rectangles exactly fitting manually the width of two MjVBs, and the height of the cross-section, see Figure 1. MnVB centres, on the other hand, are defined by fitting manually MnVBs with ovals and calculating the centres of this ovals and number.

For obtaining the *Ratio* an absolute value of the perpendicular line length connecting a MnVB centre and the baseline have been added and then divided by the baseline length.

For obtaining the *Ratio2* an absolute value of the line length connecting MnVB centre and centre of the baseline have been added and then divided by the baseline length.

For the *Salesman Ratio*, similar to the travelling salesman problem (TSP) known in mathematics [25], the shortest pathway, which goes through all the MnVB centres only once and comes back to the starting point, has been then divided by the baseline length. In order to calculate the salesman pathway the permutations of all possible pathways have been analysed, but this takes reasonable computing time (the amount of computing increase exponentially with number of points for TSP problem) only when number of points is not exceeding twelve. For the cases (like *Hewlat al Jouf* cultivar) where number of MnVB is more than twelve, the permutation process is stopped after 5 minutes computation time (for twelve points it takes less than a minute). What would mean that it is not exact solution of TSP problem and algorithms solving TSP problem does not permute all solutions, but taking into account that the difference would be very small it is irrelevant and not necessary for this particular implementation.

2) To describe a MjVB shape following parameters have been introduced:

The *Form factor* is intended to describe a deviation of a MjVB shape from a perfect circular shape, whereas *Rectangularity* describes a deviation of a MjVB shape from a rectangle. Additionally, *Aspect ratio* describes the proportional relationship between its width and its height.

An extracted shape of a MjVB has been fitted automatically with an ellipse with the smallest possible error (which has been used as a parameter *Ellipse fit residual error)*. Then *major axis a* and *minor axis b* of this ellipse have been used to describe this ellipse with parameter *Eccentricity*. So for example *Eccentricity* = 0 for a circle, *Eccentricity* = 1 for a parabola.

•

$$\mathrm{Form}\;\mathrm{factor}\;=\frac{4\pi \;\times \;\mathrm{area}\;\mathrm{of}\;\mathrm{MjVB}}{{\left(\mathrm{perimeter}\;\mathrm{of}\;\mathrm{MjVB}\right)}^{2}}$$

•

$$\mathrm{Aspect}\;\mathrm{ratio}\;=\frac{\mathrm{length}\;\mathrm{of}\;\mathrm{MjVB}}{\mathrm{width}\;\mathrm{of}\;\mathrm{MjVB}}$$

•

$$\mathrm{Rectangularity}\;=\frac{\mathrm{length}\;\mathrm{of}\;\mathrm{MjVB}\;\times \;\mathrm{width}\;\mathrm{of}\;\mathrm{MjVB}}{\mathrm{perimeter}\;\mathrm{of}\;\mathrm{MjVB}}$$

•

$$\mathrm{Eccentricity}\;=\;\sqrt{1-\frac{{\left(\mathrm{minor}\;\mathrm{axis}\;\frac{b}{2}\right)}^{2}}{{\left(\mathrm{major}\;\mathrm{axis}\;\frac{a}{2}\right)}^{2}}}$$

• *Ellipse fit residual error* = *Residual error after fitting a shape of MjVB with an ellipse*

### Artificial neural network

In order to use obtained parameters (4 of the MnVB distribution and 5 of the MjVB shape) from fluorescence images for differentiation of date palm tree cultivars, an artificial neural network (ANN) has been applied. In particular, a multilayer perceptron with bias architecture under supervised learning (backpropagation learning rule) has been used due to reportedly better results for data pattern recognition [26]. This ANN has been built and tested with the help of IBM SPSS software package ver. 19 (IBM Corp., New York, USA).

**Figure 3:**
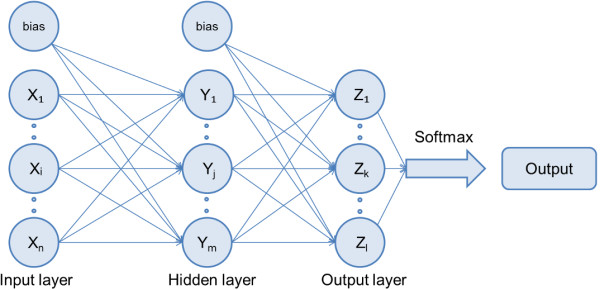
A simplified structure of the used artificial neural network.

The ANN has the following input variables – *Number of MnVB, Ratio, Ratio2, Salesman ratio, Form factor, Aspect ratio, Rectangularity, Eccentricity and Ellipse fit residual error*. The hidden layer consists of 10 nodes. As an output, the names of 5 date palm tree cultivars used in this study (*Hewlat al Jouf, Khlas, Nabot Soltan, Shishi, Um Raheem*) have been taken, see the overview of the structure in Figure 3. The *Number of MnVB* is an integer number, whereas all others are real numbers with 3 significant digits.

The hidden layer activation function has been taken as a hyperbolic tangent tan(*x*) = (*e*^*x*^ - *e*^- *x*^)/(*e*^*x*^ + *e*^- *x*^), whereas for the output layer – a softmax function $\left(y\left(x_{i}\right)=e^{x_{i}}/\sum_{j=1}^{n}e^{x_{j}}\right)$ which takes a vector of real-valued arguments and transforms it to a vector whose elements fall in the range (0, 1) and sum to 1 was used. Input variables have been rescaled with a method called standardisation, in which from each value the mean of all values is subtracted and divided by its standard deviation, (*x* - *mean*)/*std. dev*. The cross-entropy error function has been chosen due to a better network performance compared to the mean square error function [27].

A total of 85 samples were used in each ANN. Samples have been divided randomly into two groups, one group used only for the training and the other one only for the testing of the ANN, see one of the ANN’s description in Table 1. The ANN has been initialised with random initial synaptic weights. The training group has been used in an iterative process of synaptic weights adjustment in a batch mode. In this mode, only after calculation of all errors will the weight then be changed. This process provides a total error reduction after each iteration and will be stopped when no error reduction occurs anymore after weights adjustment.

**Table 1:** A summary of samples used for processing by the best ANN out of 10 in the cross-folding

		**N**	**Percentage**
Sample	Training	63	74.1%
	Testing	22	25.9%
Valid		85	100%
Excluded		0	0
Per each cultivar	*Hewlat al Jouf*	14	16.5%
	*Khlas*	22	25.9%
	*Nabot Soltan*	17	20%
	*Shishi*	20	23.5%
	*Um Raheem*	12	14.1%
Total		85	100%

## Results

After the supervised learning phase in the batch mode, and when the adjustment of synaptic weights is done, the state of the ANN is probed for a prediction of all the learning samples. The success of this process is reflected in Table 2 as a number of correct predictions in the column training. In the testing phase where the final ANN with fixed weights is tested, a number of correct predictions is reflected in the testing column.

**Table 2:** The best ANN training and testing result

**Observed**	**Percent of correct predicted**	
	**Training**	**Testing**
Hewlat al Jouf	100%	100%
Khlas	94.1%	100%
Nabot Soltan	100%	100%
Shishi	100%	100%
Um Raheem	100%	100%
Overall per cent	98.4%	100%

Taking into account that due to limited number of samples has been available for measurement, a tenfold cross-validation of ANN was performed. So that for each full learning and testing process training and test groups has been picked up again randomly from data pool. Results of each out of ten cross-validation runs presented in Table 1 and more detailed ANN performance of the best ANN out of ten in the Table 2. After ten such ANN learning testing phases, the average value was found to be 89.1%, see Table 3. Moreover ANN with radial basis layer has been used, which has been shown to have a good results in plant leave shape based recognition of plant’s species [19], but in this particular study a 10-25% lower prediction has been observed (data not shown).

**Table 3:** Overall per cent of correct predicted from tenfold cross-validation of ANN

**Cross-validation run**	**Overall per cent of correct predicted**	
	**Training**	**Testing**
1	100%	86.2%
2	100%	90.6%
3	100%	86.2%
4	100%	90.9%
5	98.4%	100%
6	92.6%	87.1%
7	100%	86.8%
8	100%	85%
9	100%	87.9%
10	100%	90.3%

The average value is 89.1%.

Variable importance analysis of the best ANN was performed with the help of IBM SPSS software in order to analyse the contribution of each used variable to the prediction rate, and is reflected in Table 4. Moreover a principle component analysis (PCA) for all the parameters was performed, which showed that there are two meaningful clusters. In the first cluster are parameters belonging to MnVB, while in the second cluster are parameters belonging to the MjVB. Reduction of the possible clusters to three shows the same parameters distribution except for *Residual Error*, which is single in the third cluster.

**Table 4:** Variable importance analysis of the best ANN

**Variable**	**Normalized importance**
Ratio	100.0%
Number	84.5%
Residual Error	77.6%
Form factor	77.5%
Ratio2	73.7%
Salesman Ratio	71.1%
Rectangularity	61.3%
Eccentricity	57.4%
Aspect ratio	52.0%

## Discussion

Many phenotypic studies of date palm tree cultivars utilize features specific to a certain time or age of a tree [14,15,28]. Analysis of fruit characteristics or protein extracts of them is unfortunately not an all-season application. Moreover, characterisation of fruits by their taste and flesh structure is often also quite subjective. In the same manner description of the whole date palm leaf or trunk is then restricted to the adult trees only. In contrast, early detection is of major interest for current date palm tree agriculture before a huge investment is made in the growth of plant of unknown properties [5].

In light of this situation a method for date palm trees differentiation should be based on features which can be readily obtained from date palm offshoots. One of these objects for feature extraction is date palm tree leaflets.

Among other types of ANN used in this work, the multilayer perceptron showed the best result and easy learning, which could be related to some correlations between extracted features. PCA revealed two or three meaningful clusters, where positive as well as negative correlations exist in clusters. Despite the fact that it is possible according to the statistical results to reduce some parameters, the application of a diminished set of features into the ANN has showed a decrease in prediction rate. These results lead to the conclusion that although parameters from MnVB or MjVB share some common information, they carry vital specific features information necessary for a better ANN performance.

As it has been mentioned before, parallel genetic studies to clarify the actual differences between cultivars would be very helpful [20,21,29]. An additional step in the direction of an industrial application could be done by possible usage of fluorescence cross section images of lower resolution, or ideally just regular light images of cross sections.

Moreover a fluorescence imaging with an artificial neural network analysis could be applied to other members of the Phoenix genus as well as for other vascular plants with linear vascular venation patterns, like maize (corn) and rice. For plants with a net-like vascular system, a different set of features need to be identified except keeping an idea of ANN usage for classification and differentiation. However the technology enabling image acquisition and handling on living trees in a plantation still remains to be developed.

## Conclusions

Overall an achieved result in prediction and differentiation of date palm tree cultivars based on the fluorescence microscopy of palm leaflets cross sections with the help of the artificial neural network was very good. The average prediction in tenfold cross-validation 89.1% and 100% in one of the best ANN’s can be considered as very promising results, in spite of only a total of 85 sample data being used in the ANN. Additionally, the fact that only 5 cultivars have been used in this study also needs to be taken into account by extrapolating this result to the general problem of date palm tree cultivars differentiation.

## Competing interests

No competing financial interests between authors exist.

## Authors’ contributions

The manuscript was written by VA who also performed the experimental and analytical work. ID and GMA reviewed and proof-edited the manuscript. DP performed technical assistance and commented on the manuscript. ATA reviewed and commented on the manuscript. All authors have read and have approved the final manuscript.

## Supplementary Material

Additional file 1: Figure S1 The DAPI-BP fluorescence image of the date palm’s *Khlas* cultivar leaflet cross section, where red line in the middle – baseline, blue line connecting centres of MnVBs – "the shortest pathway" and two red rectangles are fitting MjVBs. Click here for file

Additional file 2: Figure S2 The DAPI-BP fluorescence image of the date palm’s *Nabot Soltan* cultivar leaflet cross section, where red line in the middle – baseline, blue line connecting centres of MnVBs – "the shortest pathway" and two red rectangles are fitting MjVBs. Click here for file

Additional file 3: Figure S3 The DAPI-BP fluorescence image of the date palm’s *Shishi* cultivar leaflet cross section, where red line in the middle – baseline, blue line connecting centres of MnVBs – "the shortest pathway" and two red rectangles are fitting MjVBs. Click here for file

Additional file 4: Figure S4 The DAPI-BP fluorescence image of the date palm’s *Um Raheem* cultivar leaflet cross section, where red line in the middle – baseline, blue line connecting centres of MnVBs – "the shortest pathway" and two red rectangles are fitting MjVBs. ) Click here for file
