# Supplementary figures and images for: Phenotyping date palm varieties via leaflet cross-sectional imaging and artificial neural network application
Source: BMC Bioinformatics. 2014 Feb 24;15:55. doi: 10.1186/1471-2105-15-55 (PMC3941935; doi:10.1186/1471-2105-15-55)

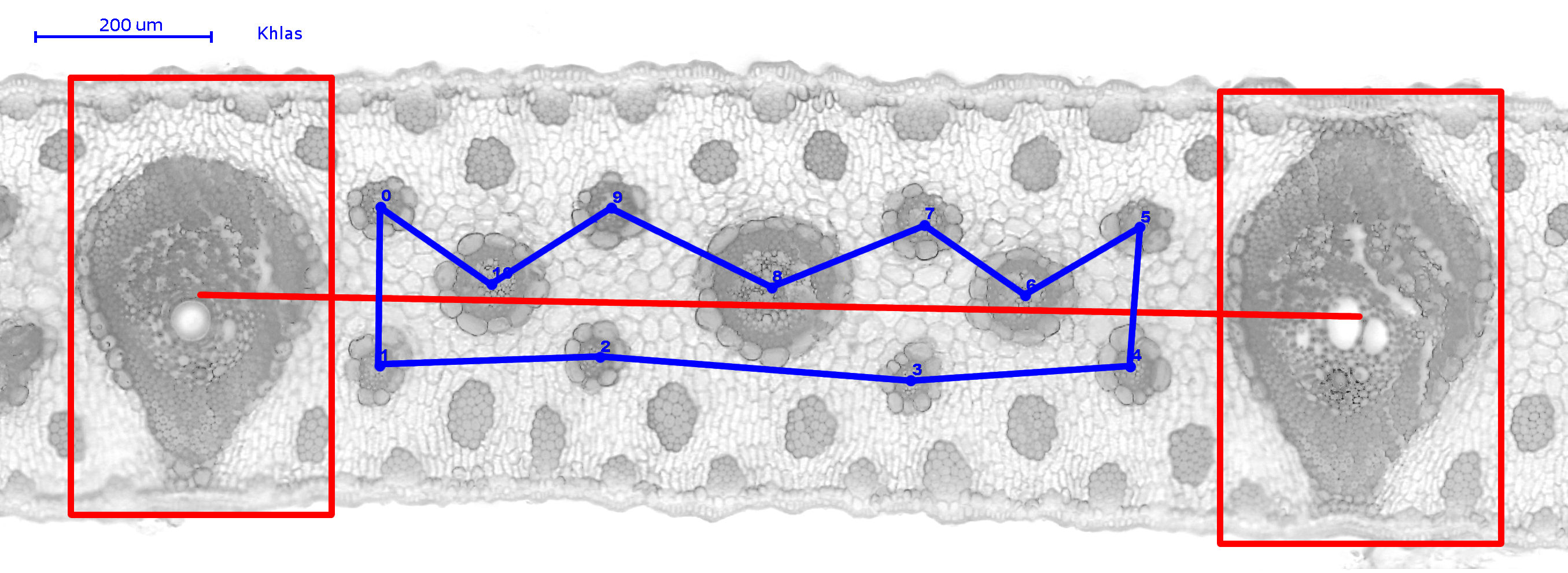

Supplement: Additional file 1: Figure S1 — The DAPI-BP fluorescence image of the date palm’s Khlas cultivar leaflet cross section, where red line in the middle – baseline, blue line connecting centres of MnVBs – "the shortest pathway" and two red rectangles are fitting MjVBs. [file 1471-2105-15-55-S1.tiff]

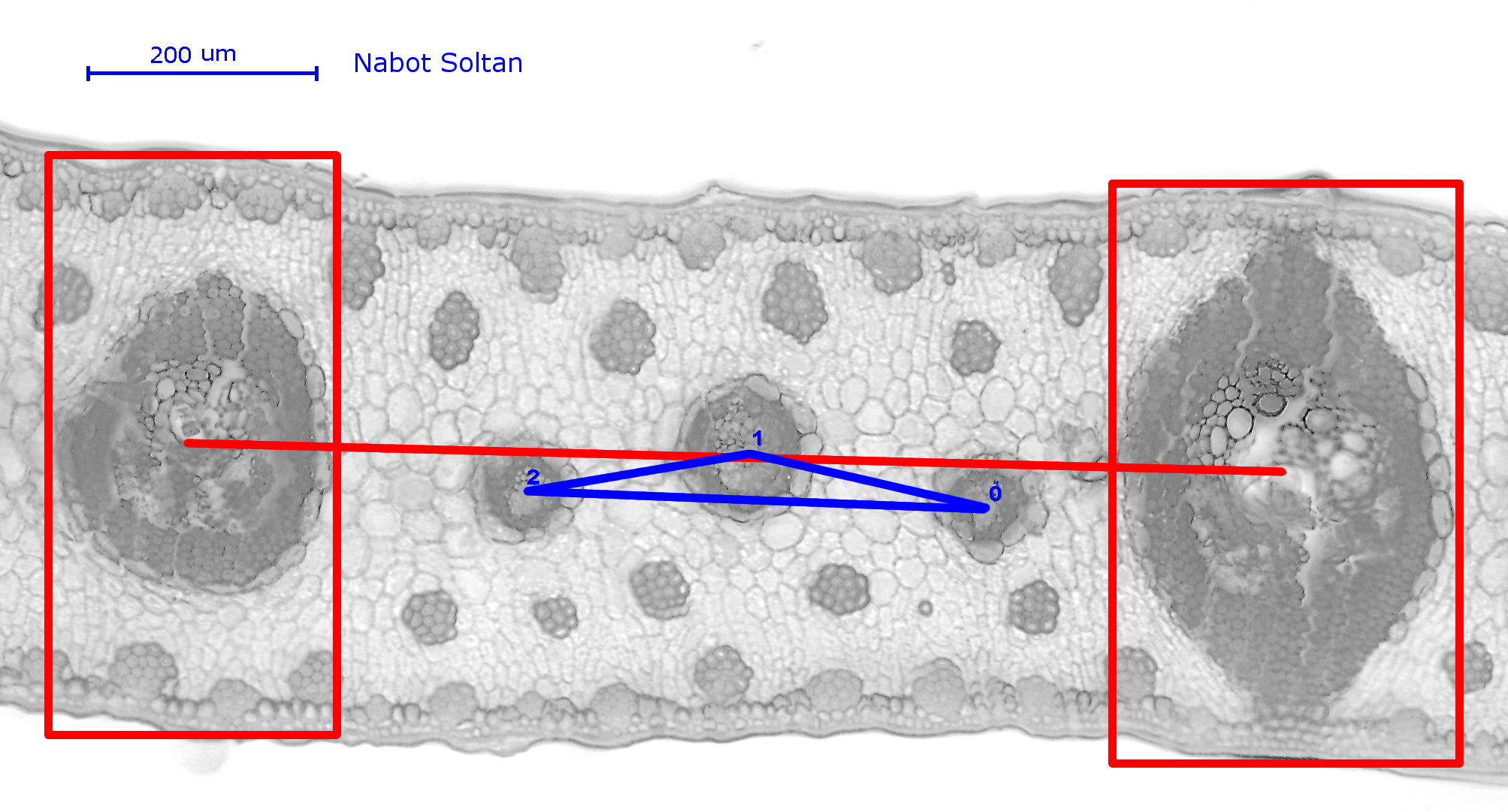

Supplement: Additional file 2: Figure S2 — The DAPI-BP fluorescence image of the date palm’s Nabot Soltan cultivar leaflet cross section, where red line in the middle – baseline, blue line connecting centres of MnVBs – "the shortest pathway" and two red rectangles are fitting MjVBs. [file 1471-2105-15-55-S2.tiff]

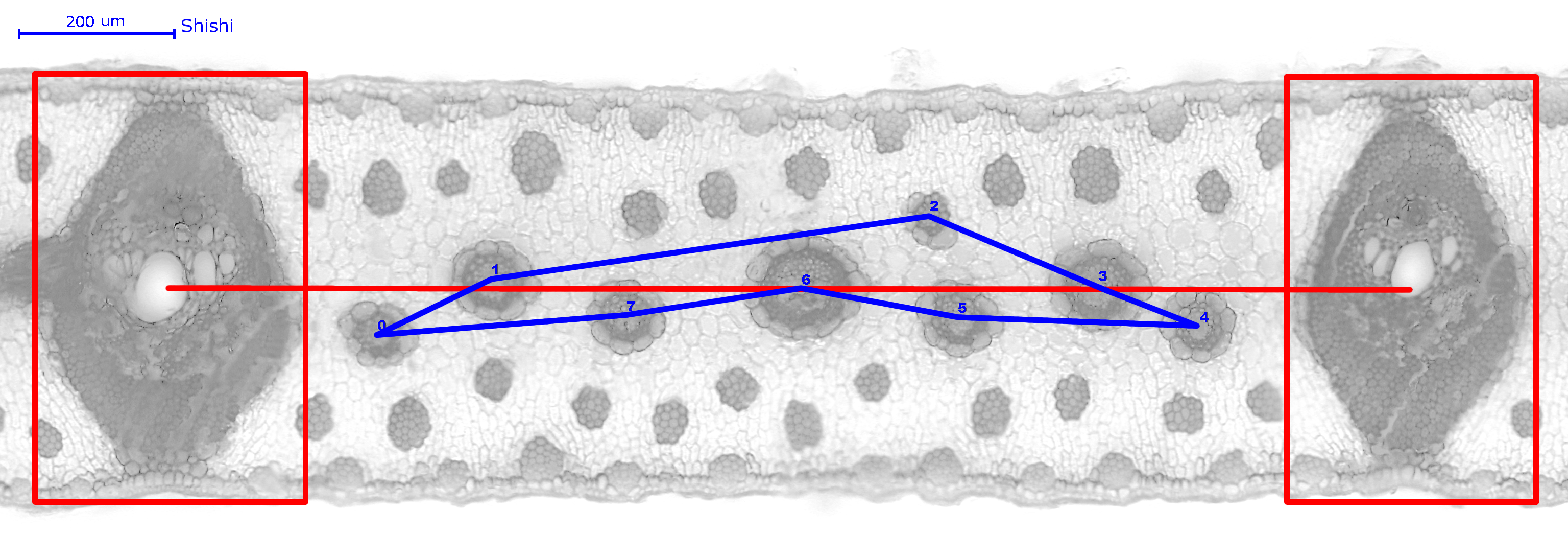

Supplement: Additional file 3: Figure S3 — The DAPI-BP fluorescence image of the date palm’s Shishi cultivar leaflet cross section, where red line in the middle – baseline, blue line connecting centres of MnVBs – "the shortest pathway" and two red rectangles are fitting MjVBs. [file 1471-2105-15-55-S3.tiff]

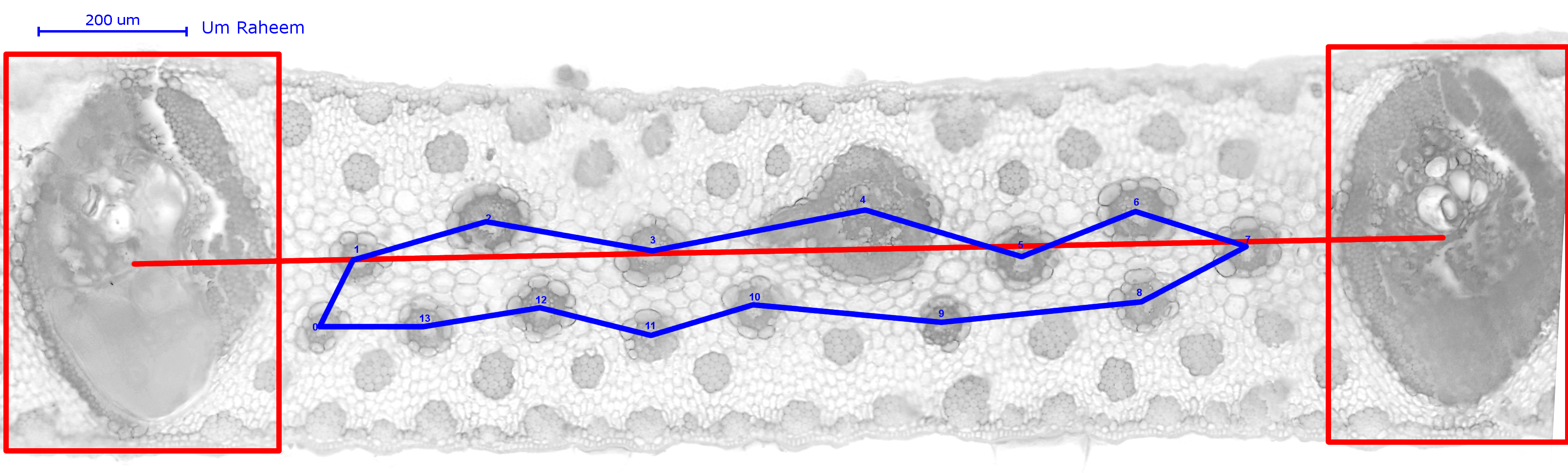

Supplement: Additional file 4: Figure S4 — The DAPI-BP fluorescence image of the date palm’s Um Raheem cultivar leaflet cross section, where red line in the middle – baseline, blue line connecting centres of MnVBs – "the shortest pathway" and two red rectangles are fitting MjVBs. ) [file 1471-2105-15-55-S4.tiff]
